# Expanded Dengue Syndrome: A Case of Subarachnoid Haemorrhage, Cranial Diabetes Insipidus, and Haemophagocytic Lymphohistiosis

**DOI:** 10.1155/2021/9932525

**Published:** 2021-10-31

**Authors:** H. M. A. U. Jayasinghe, V. Pinto, T. Jayasinghe arachchi, W. M. A. S. B. Wasala, S. Abeygunawardane, D. Dissanayake

**Affiliations:** Faculty of Medicine, University of Peradeniya, Peradeniya, Sri Lanka

## Abstract

Dengue fever is a mosquito-borne viral infection common in tropical countries with increasing incidence. The clinical manifestations can range from asymptomatic or mild infection to multiorgan failure. The latter is also called “Expanded dengue syndrome,” and it carries a high rate of mortality and morbidity. Intensive care management of such complicated cases is a challenging task for the treating physician, which requires intense monitoring and a multidisciplinary approach for decision making. We report an atypical case of an expanded dengue syndrome presented with subarachnoid haemorrhage associated with moderate thrombocytopenia, cranial diabetes insipidus, and haemophagocytic lymphohistiosis in a young healthy female patient.

## 1. Introduction

Dengue fever (DF), a mosquito-borne febrile illness caused by infection with dengue virus, is a prevalent disease in tropical and subtropical countries. The virus is transmitted to humans by infected mosquitoes, which are *Aedes aegypti* and *Aedes albopictus* [1]. The clinical manifestations of DF range from asymptomatic to multiorgan failure. Common clinical features of dengue fever are fever, leukopenia, and thrombocytopenia, and it is usually self-limiting. The diagnosis can be confirmed by the presence of IgM after about 7 days indicative of acute infection. The presence of IgG is indicative of a past infection. Rapid and early diagnosis is possible with detection of virus-produced protein NS1 antigen by ELISA in the acute phase, day 1–7 [2]. It can progress to affect the vasculature, leading to vascular leakage which defines dengue haemorrhagic fever (DHF), and it can potentially result in haemorrhagic manifestations [1, 3]. Fluid leak and depletion of intravascular volume results in severe shock syndrome and multiorgan failure and death which is also collectively named as expanded dengue syndrome [3]. Sri Lanka, being a tropical island with 2 monsoons, has a high prevalence in DF. In 2017, a total of 186,101 suspected cases and 440 dengue-related deaths were reported which is the highest in a calendar year in Sri Lanka (total population 21 million) [4]. The mortality rate of severe dengue in Asian countries is around 0.5–3.5% [1, 5]. This case report brings an unusual presentation of dengue expanded syndrome with subarachnoid haemorrhage (SAH) associated with moderate thrombocytopenia, cranial diabetes insipidus (CDI), and haemophagocytic lymphohistiosis (HLH) in a young healthy female patient which highlights the extent of the complicated dengue challenging the clinicians.

## 2. Case History

A 41-year-old previously healthy female was admitted to a local hospital in Sri Lanka with a history of fever (38.9°C) associated with myalgia, arthralgia, headache, and abdominal pain for two days. She was diagnosed as DF with a positive Dengue NS 1 antigen test on day 3. She was managed for five days in the medical ward with close monitoring and cautious fluid management as per Sri Lankan national guideline on management of DF. Her condition remained stable throughout the initial five days without evidence of leaking or bleeding with a dropping but above 100 × 10^9^/l platelet count. However, on the 6^th^ day of fever, she was diagnosed to have entered the critical phase with clinical evidence of leaking and her packed cell volume (PCV) rose to 50. By this time, platelet count had also dropped to 57 × 10^9^/l. During the same episode, her behavior was altered and she developed an episode of generalized tonic clonic convulsion that resolved spontaneously in two minutes. As her conscious level further deteriorated to a GCS of six, she was intubated and neuroprotective ventilation was commenced suspecting an intracranial haemorrhage. She was transferred to a tertiary-care unit, for CT brain and further specialized management. She progressed to a circulatory shock state despite several crystalloid and colloid boluses, as per guidelines. Meanwhile, serum calcium, sugar, and acidosis were corrected. Noradrenalin infusion was commenced to maintain mean arterial pressure more than 70 mmHg. Although the clinical picture was strongly suggestive of dengue haemorrhagic fever with leaking, there was no definite evidence of significant fluid collection in the lung bases or abdomen on ultrasound examination except for a mild gall bladder wall oedema. Possibility of a significant bleeding was less likely as her PCV and hemoglobin were rising. The bedside 2D echocardiogram reveled a low ejection fraction of 30–35% with globally hypokinetic left ventricle. T inversions and ST changes were evident on ECG and troponin I titre was elevated, all pointing towards a diagnosis of dengue myocarditis.

CT brain revealed an SAH of Fishers grade 3 with diffuse cerebral oedema. Because of the high risk of bleeding with a persistently low platelet count of 30–40 × 10^9^/l and haemodynamic instability, decision was taken to manage conservatively without performing a decompressive craniotomy. Hence, neuroprotective ventilation was continued with the implementation of intracranial pressure-reducing strategies.

Due to SAH, platelet count needed to be maintained close to 100 × 10^9^ mm^3^. Despite platelet transfusion, the platelet count remained below 40 × 10^9^/l. There was no evidence in the blood picture to suggest disseminated intravascular coagulation or thrombotic thrombocytopenic purpura. Hence, she was investigated on the line of HLH. Serum ferritin was 1500 *μ*g/l, and LDH was 1247 *μ*/l. Bone marrow biopsy confirmed HLH. She was treated with immunoglobulin 20 g daily and high-dose dexamethasone as for HLH.

Even though she initially developed stage 1 acute kidney injury, on the second day of ICU admission, she developed polyuria (3.2 ml/kg/hr), with high serum osmolality and urinary sodium of 160 mmol/l. In the context of raised ICP following SAH, CDI was diagnosed along with urinary studies. Polyuria and hypernatremia further complicated the fluid management making bedside 2D echo and point-of-care ultrasound scanning a valuable tool to guide fluid therapy. Desmopressin nasal puffs were also used to control CDI.

Her condition continued to deteriorate despite the active multidisciplinary management. She expired on the third day of admission to the tertiary-care unit.

## 3. Discussion

This patient presented with DF which progressed to DHF and circulatory shock got complicated with an SAH, CDI, and HLH ending in a fatality. This case highlights several important unusual presentations and challenges in the management of dengue.

PCV rise from 41.1 to 50 with concurrent rise of haemoglobin from 13.9 to 16.4 g/dl on day six indicates fluid leakage and development of DHF. The presence of dengue myocarditis was also contributary to the refractory shock. There was no evidence to suggest bleeding as a cause for her refractory shock as her haematocrit and hemoglobin level remained high throughout with no identifiable external bleeding. DF and DHF are associated with a varying degree of thrombocytopenia. Despite platelet count dropping even less than 10 × 10^9^/mm^3^ on most occasions, these patients do not develop significant bleeding manifestations except for minor such as gum bleeding or bruising. However, the exact prevalence of these is yet to be described [3].

Neurological complications in dengue fever occur in less than 1% of population, and these can be due to either direct infection by the virus, haemorrhagic complications, or because of multiorgan failure [6]. Dengue-related encephalopathy, encephalitis, aseptic meningitis, intracranial haemorrhages, mononeuropathies and polyneuropathies, Guillain–Barre syndrome, and myelitis have been reported [5]. This case reveals an SAH with cranial DI, which is a rare occurrence in DHF. This occurred when the platelet count was in the range of 57–44 × 10^9^/dl, which is not significantly low to cause a spontaneous intracranial bleeding. The absence of any other bleeding manifestations raises the suspicion of cerebral pathologies such as AV malformation or aneurysm; however, her past medical history or family history was not suggestive of any. One case report is available that reveals a subdural haematoma and diffuse intracranial bleeding complicated with cranial DI [6]. A review of dengue patients with SAH by Wiwanitkit states that most of the reported cases had delayed diagnosis and this probably led to high mortality [7]. We found another case report of SAH in dengue fever with complete recovery following correction of coagulopathy and decompressive craniotomy [8]. A review of 22 patients of intracranial bleeding (ICH) in dengue fever by Sam et al. found that platelet counts on diagnosis of ICH ranged from 15 to 100 × 10^9^/L. They further state that patients with lower platelet levels do not necessarily have worse outcomes and that higher platelet levels do not seem to protect patients from fatal ICH [9].

Development of intracranial bleeding with moderate thrombocytopenia warrants a search for other causes of bleeding in dengue which could be the use of nonsteroidal anti-inflammatory drugs, DIC due to multiorgan failure, or coagulopathy induced by liver failure, which were not evident in this case. Bleeding with normal clotting profile may be explained by defects in platelet functions which are known to occur in dengue infection [6]. However, we do not carry out platelet function tests in our routine clinical practice.

HLH is a rare complication of DF, and only a few cases are reported [10]. It is characterized by the activation of macrophages and cytokine release resulting in an immune dysregulation. Patients with HLH show bicytopenia, coagulopathy, liver dysfunction, hyperferritinemia, and elevated triglycerides and lactate dehydrogenase. Bone marrow biopsy reveals evidence of infiltration by activated macrophages in HLH [8]. HLH is diagnosed by the HLH-2004 protocol. Raised ferritin >10000 ng/ml is recognized to be 98% sensitive and 96% specific for HLH; hence, it is a useful screening tool for early detection of HLH, triggering further investigations [11]. However, clinical conditions such as sepsis or systemic inflammatory response syndromes can show similar characteristics [10]. HLH is a known association with poor prognosis. Treatment of HLH involves treatment of the provocative illness if secondary, use of immune-suppressive and modulatory agents, and subsequent stem-cell transplantation. The agents commonly used include dexamethasone, methylprednisolone, methotrexate, intravenous immunoglobulin, and cyclosporine A [10, 11].

## 4. Conclusions

Expanded dengue syndrome with SAH, cranial DI, and immunological complications such as HLH and multiorgan failure is extremely rare and predicts a poor prognosis. The pathophysiology behind ICH in dengue infection is complex and probably multifactorial caused by coagulopathy, platelet dysfunction, thrombocytopenia, and vasculopathy; hence, bleeding has to be considered even with a platelet count of more than 50 × 10^9^/L. Explanation for the exact mechanism for ICH in only a rare minority is yet to be proven. It can be postulated that probably an immunological response be the initiation for activation of HLH and intracranial haemorrhages in this case. Given the rising spread of dengue epidemic in tropical and subtropical counties, high degree of suspicion, prompt recognition, and aggressive management of atypical, uncommon, but sinister dengue syndromes are crucial for successful outcome.
